# Percutaneous endoscopic interlaminar discectomy of L5–S1 disc herniation: a comparison between intermittent endoscopy technique and full endoscopy technique

**DOI:** 10.1186/s13018-017-0662-4

**Published:** 2017-10-30

**Authors:** Hongyu Song, Wenhao Hu, Zhongguo Liu, Yongyu Hao, Xuesong Zhang

**Affiliations:** 1 Department of Orthopaedics, General Hospital of People’s Liberation Army, Beijing, People’s Republic of China; 2 Department of Orthopaedics, The Third Hospital of Xiamen, Xiamen, People’s Republic of China

**Keywords:** Percutaneous endoscopic lumbar discectomy, Interlaminar approach, Clinical outcome, Herniated nucleus pulpous, Full endoscopy, Intermittent endoscopy, MacNab criteria, Intracanalicular disc herniation

## Abstract

**Background:**

Percutaneous endoscopic laminar discectomy is a typical minimally invasive discectomy operation that is classified into the percutaneous endoscopic transforaminal discectomy and the percutaneous endoscopic interlaminar discectomy. Based on whether the surgeon chooses to deal with the ligamentum flavum under endoscope guidance, percutaneous endoscopic discectomy by the interlaminar approach can be performed with a full endoscope technique with the intermittent endoscope technique. To our knowledge, there is no study comparing these two techniques in regard to their surgical effects and advantages. Therefore, we conducted this study to compare the cost, safety, and efficacy between the intermittent endoscopy technique and full endoscopy technique of endoscopic interlaminar lumbar discectomy at the L5–S1 level.

**Methods:**

From September 2014 to March 2015, a total of 126 patients with radiculopathy due to L5–S1 disc herniation who were treated by a full endoscopy technique (65 patients) or intermittent endoscopy technique (61 patients) were included. Relevant data, such as duration time of the operation, hospitalization expenses, postoperative bed rest time, length of hospitalization, and complication rates, were recorded. Clinical outcomes were assessed by the visual analog scale score, modified MacNab criteria, and Oswestry disability index.

**Results:**

In the full endoscope (FE) group, the mean duration time of surgery was 75.0 ± 11.9 min. The postoperative bed rest time was 6.5 ± 1.1 h, length of hospitalization was 3.8 ± 1.1 days, and complication rate was 7.69%. In the intermittent endoscopy (IE) group, the mean duration time of surgery was 43.0 ± 16.4 min. The postoperative bed rest time was 5.0 ± 1.1 h, length of hospitalization was 3.6 ± 1.2 days, and complication rate was 6.60%. The average hospitalization expenses of the FE group and IE group, respectively, were 32,069 ± 1086 RMB and 22,665 ± 899 RMB. There were significant differences in the surgical duration and hospitalization expenses (*P* < 0.01), but no differences between the two groups in postoperative bed rest time, length of hospitalization, or complication rates (*P* > 0.05). The postoperative Oswestry disability index and VAS were clearly improved in both groups compared with those of preoperation (*P* < 0.01). These two procedures have the same clinical outcomes (*P* > 0.05).

**Conclusions:**

Both the full endoscopy technique and intermittent endoscopy technique achieved good outcomes, whereas the intermittent endoscopy technique is a more effective option for a shorter duration surgery and lower hospitalization expenses.

## Background

Minimally invasive techniques accurately targeting pathological tissue are being developed for spine surgery. Percutaneous endoscopic laminar discectomy (PELD) is a typical representative minimally invasive discectomy surgery that can be classified into percutaneous endoscopic transforaminal discectomy (PETD) and percutaneous endoscopic interlaminar discectomy (PEID), according to the approach to the herniation disc materials. Previous studies have suggested that the transforaminal approach is difficult because the high iliac crest, large L5 transverse process, large facet joint, and narrowed disc space might limit clinical access to the L5–S1 disc space [1–3]. Additionally, PEID has advantages, including a faster puncture orientation, shorter operation time, and less intraoperative radiation exposure than PETD to treat L5–S1 disc herniation [4]. PEID is widely accepted to treat L5–S1 disc herniation because of its minimal surgical trauma and similar approach to open surgery. Depending on the method of ligamentum flavum management [5], PEID is mainly performed with a full endoscope technique or an intermittent endoscopy technique. The most different point between full endoscopy technique and intermittent endoscopy technique is the method to enter into the epidural space: full endoscope technique was first described by Ruetten [8], who put a working catheter on the surface of the ligamentum flavum through a dilator and broke the ligamentum flavum under endoscopic direct vision. Intermittent endoscopy technique involves inserting a wire directly into the disc space, split by sequential insertion of serial dilators to approach the epidural space under fluoroscopic guidance by patient’s responses. We performed this study because few existing studies have systematically compared these two techniques. In this retrospective study, we tried comparing the surgical duration, hospitalization expenses, complications, and surgical effects between the two techniques.

## Methods

The study was approved by the Ethics Committee of the General Hospital of People’s Liberation Army, and all patients provided informed consent. All procedures involving human participants were performed in accordance with the Declaration of Helsinki. From September 2014 to March 2015, a total of 126 patients with radiculopathy due to L5–S1 disc herniation who were treated by PEID using the full endoscopy technique (65 patients) or intermittent endoscopy technique (61 patients) were included in this retrospective analysis. Relevant data, such as the surgical duration, hospitalization expenses, postoperative bed rest time, hospitalization duration, and complication rates, of these two groups were recorded. Clinical outcomes were assessed by the visual analog scale (VAS) score, modified MacNab criteria, and Oswestry disability index (ODI) with a follow-up period of more than 2 years.

The inclusion criteria in this study were as follows: (1) patients with symptomatic radiating leg pain that was more prevalent than back pain, positive straight leg-raising test; (2) computed tomography and magnetic resonance imaging suggesting a single level disc herniation at the L5–S1 level correlated with the clinical findings; (3) regular expectant treatment for 6 weeks without any significant relief being achieved; and (4) no previous lumbar surgical history at the same level. The exclusion criteria were as follows: (1) intracanalicular stenosis, (2) segmental instability, (3) recurrent disc herniation at the same level, and (4) coexisting pathological, conditions such as infection, tumor, or fracture.

### Surgical procedures

**Fig. 1:**
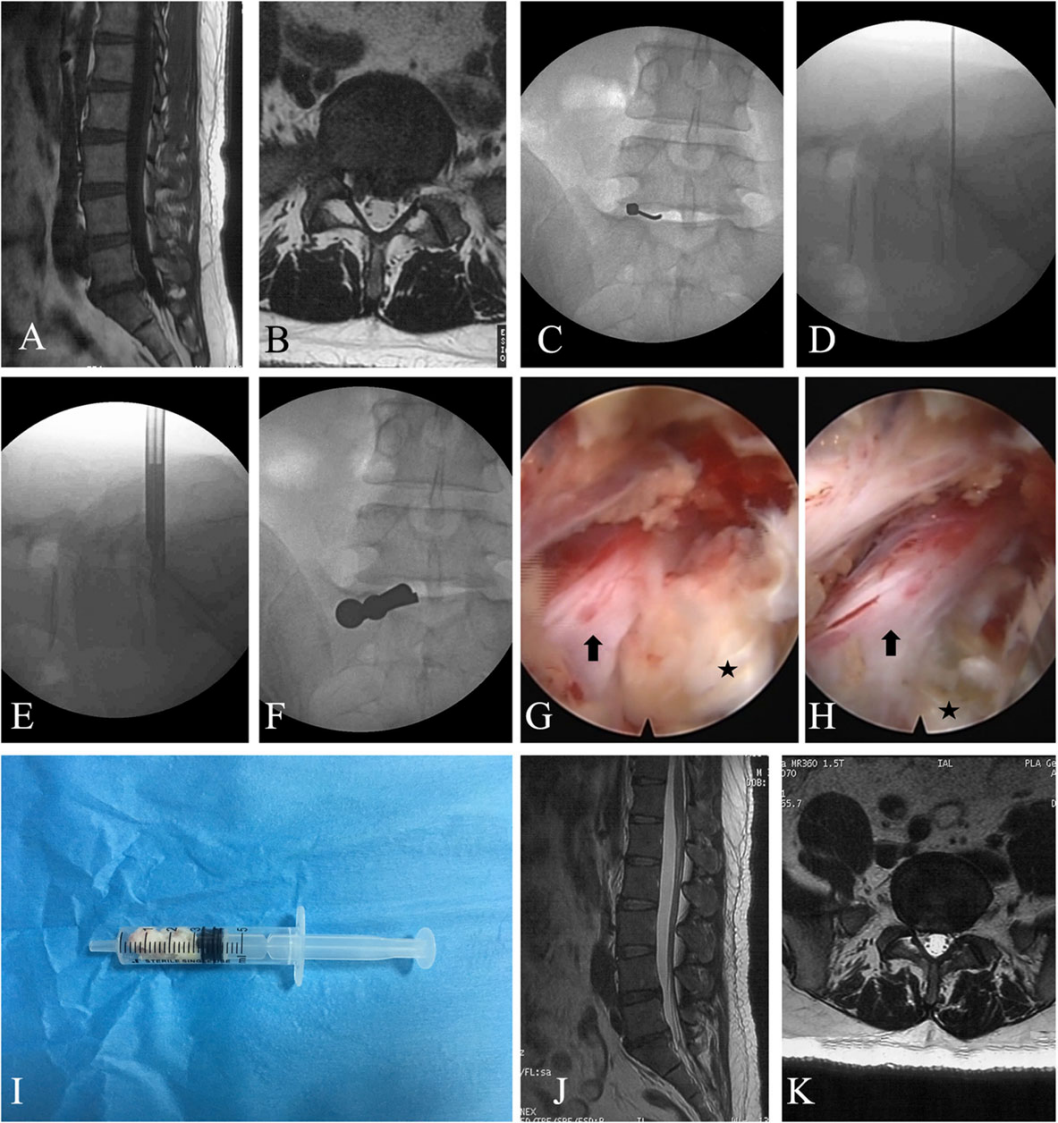
Percutaneous endoscopic interlaminar discectomy using intermittent endoscopy technique for a 40-year-old male patient with L5–S1 disc herniation. **a**, **b** Preoperative MRI image showing compression of dura and right S1 nerve root by disc herniation. **c**, **d** Place the needle into the disc. Intraoperative x-ray showing the placement of the dilator. **e**, **f** The working cannula was rotated in, and the location was confirmed with C-arm fluoroscopy before discectomy. **g** Intraoperative view in interlaminar access with S1 nerve root (arrow) and the herniation (star). **h** Intraoperative view in interlaminar access with S1 nerve root (arrow), ruptured annulus fibrosus (star) after removal of the herniation. **i** Disc pulposus. **j**, **k** 31 month postoperative MRI showing removal and good decompression of nerve root and dura

1) Full endoscope technique: Patients were placed in the prone position on a bow-type frame solidly attached to the operating table under general anesthesia to make the interlaminar window wider. Under anterior posterior fluoroscopy, the lumbar process at the surgical segment was superficially located to mark the posterior midline. The skin incision was made as close to medial in the craniocaudal middle of the interlaminar window as possible. A 0.8-cm incision was made with a sharp scalpel at the entry point of the puncture needle. A dilator, 7.0 mm in outer diameter, was bluntly inserted in the lateral edge of the interlaminar window. After endoscope insertion, the obstructing muscle and fat were removed to visualize the ligamentum flavum. The ligamentum flavum was vertically split by a dissector, and the work channel was inserted into the epidural space so the bevel, fat tissue, dural sac, and S1 roots could be identified. Rotating and inserting the work channel into the shoulder or axilla of the nerve root under direct visual control and continuous irrigation, the channel was used as a nerve hook. Then, the extruded disc could be seen, and sequestrectomy was performed. The completeness of decompression was assessed. It was necessary to reconfirm if there were any remaining disc fragments in the S1 root. Annulus fissure coagulation and hemostasis were performed using the bipolar radiofrequency at 15 W for coagulation. After removal of the scope, skin was sutured with a single stitch.

2) Intermittent endoscopy technique: Patients were placed in the prone position on a bow-type frame solidly attached to the operating table to widen the interlaminar window. The operating table had a horizontal tilt of 15 degrees to the surgical side. Under anterior-posterior fluoroscopy guidance, the posterior midline at the L5–S1 segment was superficially marked. Lignocaine (0.5%) was locally injected when necessary. A puncture needle was inserted 1 cm lateral to the posterior median line. The puncture needle was advanced in a paramedian approach with the use of the loss-of-resistance to air technique. Continuous feedback from the patient is a very important method to avoid neural injury. If the patient experienced severe radicular pain, the puncture needle was pulled out and the direction of the piercing was changed. After negative aspiration for cerebrospinal fluid and blood, confirming that the needle reached the epidural space, lignocaine (2 mL 0.5%) was injected to “flow away” the nerve root and dural capsule. Then, visualized in the lateral view, the needle was punctured into the L5–S1 disc. A 0.8-cm incision was made with a sharp scalpel at the entry point of the puncture needle. A dilator was inserted into the interlaminar space through the ligamentum flavum. A working cannula was rotated along the dilator to the location and was confirmed with G-arm fluoroscopy. The endoscope was then placed. Under endoscopy, the extruded disc could be seen, and sequestrectomy was performed. With upward rotation of the working cannula, the epidural fat, nerve root, and dural capsule were exposed. Then, the nerve root was explored exteriorly upward, and downward by adjusting the working cannula. Good mobility of the nerve root and good pulse of the intra-canal dural capsule by reexamination suggested complete decompression. Annulus fissure coagulation and hemostasis were undertaken by bipolar radiofrequency at 15 W for coagulation. After removing the scope, the skin was sutured with a single stitch (Fig. 1).

All data were analyzed with SPSS software, version 19.0 (SPSS, Chicago, IL) for Windows. Data are presented as the means ± standard deviation (SD). The intergroup surgical time, anesthesia expenditure, postoperative bed rest time, and hospitalization time sample means were compared with a *t* test. The intragroup preoperative and postoperative VAS and ODI were compared with a paired *t* test. Intergroup satisfaction scores and complications used the *x*2 test. *P* < 0.05 was set as a significant level.

## Results

This study was conducted to evaluate and compare the effects of two different PELD techniques and included a total of 126 patients. The average length of the study period was 27 months. Patients were grouped to undergo either of the surgical procedures. There were 36 males and 29 females in the FE group (mean age = 52.50 ± 9.94 years) and 33 males and 28 females in the IE group (mean age = 53.50 ± 9.09 years). As shown in Table 1, there were no statistically significant differences between the two groups in age, gender, or pain duration. All patients completed the daytime surgery successfully without dislocation, dural rupture, nerve root injuries, or infections. All patients were followed up without loss for at least 27 months via phone or outpatient rechecks.

**Table 1:** General characteristics of the subjects

	FE group	IE group	*P* value
Gender M/F	36/29	33/28	> 0.05
Age (years)	52.50 ± 9.94	53.50 ± 9.09	> 0.05
Types			> 0.05
Central	24	20	
Paracentral	20	19	
Prolapsus/sequestered	21	22	
Symptoms			> 0.05
Low back pain	63	60	
Leg pain	65	61	
Signs			> 0.05
Lasegue test (+)	61	58	
Enhanced Lasegue test (+)	60	56	
Paresthesia in lower leg	64	60	
Lower extremity weakness			> 0.05
Peroneus longus and brevis	34	31	
Gastrocnemius-soleus complex	32	36	
Gluteus maximus	11	9	
Achilles tendon reflex weakness	40	37	
Pain duration (years)	2.6 ± 2.4	2.7 ± 2.6	> 0.05

In the FE group, the mean duration time of surgery was 75.0 ± 11.9 min. The postoperative bed rest time was 6.5 ± 1.1 h, and the length of hospitalization was 3.8 ± 1.1 days. In the IE group, the mean duration time of surgery was 43.0 ± 16.4 min, postoperative bed rest time was 5.0 ± 1.1 h, and length of hospitalization was 3.6 ± 1.2 days. The average hospitalization expenses of the FE group and IE group, respectively, were 32,069 ± 1086 RMB and 22,665 ± 899 RMB (Table 2). There were significant differences in the surgical duration and hospitalization expenses (*P* < 0.01). There was no difference between the two groups in the bed rest time and length of hospitalization (*P* > 0.05).

**Table 2:** Surgical results

	Operation time (min)	Postoperative bed time (h)	Hospitalization time (days)	Hospitalization expenses (RMB)
FE group	75.0 ± 11.9^*^	6.2 ± 1.3	3.8 ± 1.1	32,069 ± 1086^*^
IE group	43.0 ± 16.4	5.7 ± 1.1	3.6 ± 1.2	22,665 ± 899

^*^ *P* < 0.01 versus IE group

There was no significant difference between the two groups in follow-up time. According to the VAS and ODI scores, there was a significant (*P* < 0.01) improvement in leg pain and daily activities scoring using the VAS and ODI in the two groups. The differences in the results were not significant between the two groups (*P* > 0.05) (Table 3).

**Table 3:** Clinical outcome including VAS pain scores, ODI scores, and North American Spine Society Instrument scores

	FE group	IE group	*P* value
Follow-up period (months)	27.5	27.8	> 0.05
Preoperative VAS	8.23 ± 0.93	8.25 ± 1.05	> 0.05
Last follow-up VAS	2.60 ± 1.60	2.40 ± 1.50	> 0.05
Preoperative ODI (%)	62.0 ± 14.2	63.9 ± 15.7	> 0.05
Last follow-up ODI (%)	7.60 ± 1.60	7.60 ± 1.60	> 0.05
MacNab evaluation			
Excellence	52	48	
Good	9	9	
Fair	3	4	
Poor	1	0	
Excellence/good rate	90.80%	91.40%	> 0.05
Complications			
Fragment omissions	0	0	
Nerve root injury	0	0	
Postoperative dysesthesia	4	3	
Recurrent disc herniation	1	0	
Rhachiaesthesia	0	1	
Complication rate	7.69%	6.60%	> 0.05

For the modified MacNab criteria, there were 52 excellent cases, nine good cases, three fair cases, and one poor case, with an excellent/good ratio of 93.8% in the FE group. In the IE group, there were 48 excellent cases, nine good cases, four fair cases, and zero poor cases, with an excellent/good ratio of 93.4%. There was no significant difference in the excellent/good ratio between the two groups (*P* > 0.05). Nine patients experienced complications during the study (complication rate of 7.14%). No serious complications, such as dural tear or nerve root injury, occurred. There was no significant difference in the complication rate between the two groups (*P* > 0.05); four patients in the FE group (two males, two females) and three patients (three males) in the PETD IE group developed postoperative dysesthesia. Patients’ symptoms were rehabilitated after conservative treatment from 4 to 6 weeks. One male patient had a recurrent disc herniation 3 months after surgery in the FE group and underwent conventional open revision surgery in a local hospital. One female patient in the IE group experienced rhachiaesthesia when the surgeon injected 0.5% lignocaine into the epidural space at the beginning of surgery; the patient felt chest distress and dyspnoea, with decreasing blood pressure. The patient was turned to the dorsal position, and oxygen was delivered at the rate of 3 L/min via face mask. After approximately 10 min, when she was feeling better, surgery was restarted. We assumed that the tip of the needle was inserted into the dural capsule when injecting lignocaine. Luckily, no serious consequences occurred because the dose of anesthetic drug was very small (less than 5 mL).

## Discussion

Although conventional open surgery has been considered the gold standard to manage lumbar disc herniation [6], there remains the problem of soft tissue damage of the surrounding muscles, ligaments, and facet joints. For decades, minimally invasive techniques have been widely used to minimize the disadvantages of open surgical procedures. Since Yeung et al. [7] developed the spinal endoscopic YESS system, percutaneous endoscopic discectomy has become one of the most common minimally invasive spinal surgeries. Many studies have [8, 9] indicated that the PELD technique has advantages, such as a smaller incision, less damage to soft tissues, decreased blood loss, shorter length of hospitalization, and expedited return to work, compared with the open technique, but has the same therapeutic effect as conventional surgery. According to the approach, PELD can be classified into percutaneous endoscopic transforaminal discectomy (PETD) and percutaneous endoscopic interlaminar discectomy (PEID). PETD shows good results for nerve root decompression, with low complication rates in herniated lumbar disc removal [4, 7–9]. However, the transforaminal approach to the L5–S1 disc space is difficult because a high iliac crest, large L5 transverse process, large facet joint, or narrowed disc space and neuroforamen all serve to limit clinical access [10, 11]. There is less laminar overhang of the L5 vertebra, the interlaminar distance was greatest at the L5–S1 level, and the width of the interlaminar space was also at a maximum at 31 mm (range 21–40 mm) [12]. In addition, the depth of this recess up to the dura measures 3 to 4 mm and is occupied by epidural fat, which forms the working space for interlaminar endoscopy upon entry [13]. The various anatomic features of the L5–S1 interlaminar space make the interlaminar approach possible. Since PEID was introduced to treat L5–S1 disc herniation, it has been widely used due to its minimal surgical trauma and approach similar to open surgery. Hongfei Nie et al. [4] performed a random prospective study on the two approaches in treating patients with L5–S1 lumbar disc herniation and found that the PEID approach avoided blockade of the iliac crest and had advantages in terms of faster puncture localization, shorter surgical time, and less radiation exposure and was more suitable for a prolapsed and sequestered L5–S1 disc herniation.

The most important factor to consider for the interlaminar approach is management of the ligamentum flavum. In 2005, Doctor Choi [14] introduced an intermittent endoscopy technique of PEID under local anesthesia; this method involved inserting a wire directly into the disc space, split by sequential insertion of serial dilators to approach the epidural space under fluoroscopic guidance by patient’s responses. He reported 67 cases operated for a prolapsed intervertebral disc at the L5–S1 level with the intermittent endoscopy technique, and fifty-nine patients (90.8%) showed excellent to good results with a follow-up period of more than 18 months. Another surgical technique (full endoscope technique) was reported by Ruetten [15, 16]; this method involved placing the working catheter on the surface of the ligamentum flavum through the dilator and breaking the ligamentum flavum under endoscopic direct vision. Jung-Sup Lee et al. [5] thought that the second method seemed safer, with excision and surgery under visual control. However, there is no study comparing these two PEID techniques in regard to their surgical effects and advantages.

In the current study, we compared the clinical results, technique feasibility, safety, and efficacy of endoscopic interlaminar discectomy at L5–S1 with the full endoscopy technique and intermittent endoscopy technique. The results indicated that the IE group experienced significantly less operation time and hospitalization expenses than the FE group. This advantage of the IE technique might depend on the following facts: (i) the intermittent endoscopy technique directly inserts a dilator into the interlaminar space, through the ligamentum flavum with continuous feedback from the patient [17]. The full endoscopy technique needs to recognize and split the ligamentum flavum under endoscopy. It was difficult to manage the ligamentum flavum in a limited visual field and operation space. (ii) The intermittent endoscopy technique is more effective and economic due to the use of local anesthesia and less anatomical trauma; this method results in less damage to the ligamentum flavum because the operation is performed with ligamentum flavum splitting. The full endoscopy technique could not prevent injury to the ligamentum flavum. (iii) The intermittent endoscopy technique accurately targets the disc hernia, easily finding the extruded disc under endoscopy. It is important and necessary to make a preoperative decision. Different types of herniation require different skin entry points; if the herniation is located just ventral to the nerve root on the shoulder region, there are no safe spaces in the axillar at that time. Therefore, we made a decision to perform the shoulder approach for those cases. On the other hand, these cases usually had a safe area at shoulder region [14].

We did not show a definite difference in outcomes for the VAS and ODI scores between the FE group and IE group. Both techniques achieved good outcomes and high patient satisfaction. No serious complication, such as dural tear or nerve root injury, occurred, and there was no significant difference in the complication rate between the two groups, indicating that both methods were safe and feasible. We prefer the intermittent endoscopy technique because it is more effective and economic, and the avoidance of intraoperative nerve injury is easier due to intraoperative feedback from patients.

## Conclusions

Both the full endoscopy technique and intermittent endoscopy technique achieve good outcomes and high patient satisfaction, and the intermittent endoscopy technique is a more effective option because of its shorter surgical duration and lower hospitalization expenses.

## Abbreviations

FE: Full endoscope
IE: Intermittent endoscopy
ODI: Oswestry disability index
PEID: Percutaneous endoscopic interlaminar discectomy
PELD: Percutaneous endoscopic laminar discectomy
PETD: Percutaneous endoscopic transforaminal discectomy
VAS: Visual analog scale
